# Effect of oral administration of *Bacillus coagulans* B37 and *Bacillus pumilus* B9 strains on fecal coliforms, *Lactobacillus* and *Bacillus* spp. in rat animal model

**DOI:** 10.14202/vetworld.2016.766-772

**Published:** 2016-07-26

**Authors:** Lopamudra Haldar, D. N. Gandhi

**Affiliations:** 1 Department of Basic Sciences, Faculty of Science and Technology, ICFAI University, Kamalghat - 799 210, Tripura, India; 2 Dairy Microbiology Division, National Dairy Research Institute, Karnal - 132 001, Haryana, India

**Keywords:** acid salt tolerance, antibacterial activity, *Bacillus coagulans*, *Bacillus pumilus*, bile salt tolerance, probiotics

## Abstract

**Aim:**

To investigate the effect of oral administration of two *Bacillus* strains on fecal coliforms, *Lactobacillus* and *Bacillus* spp. in rat animal model.

**Materials and Methods:**

An *in vivo* experiment was conducted for 49-day period on 36 adult male albino Wister rats divided equally into to four groups. After 7-day adaptation period, one group (T1) was fed on sterile skim milk along with basal diet for the next 28 days. Second (T2) and (T3) groups received spore biomass of *Bacillus coagulans* B37 and *Bacillus pumilus* B9, respectively, suspended in sterilized skim milk at 8-9 log colony-forming units/ml plus basal diet for 28 days, while control group (T4) was supplied with clean water along with basal diet. There was a 14-day post-treatment period. A total of 288 fecal samples (8 fecal collections per rat) were collected at every 7-day interval starting from 0 to 49 days and subjected to the enumeration of the counts of coliforms and lactobacilli and *Bacillus* spores using respective agar media. *In vitro* acid and bile tolerance tests on both the strains were performed.

**Results:**

The rats those (T2 and T3) received either *B. coagulans* B37 or *B. pumilus* B9 spore along with non-fermented skim milk showed decrease (p<0.01) in fecal coliform counts and increase (p<0.05) in both fecal lactobacilli and *Bacillus* spore counts as compared to the control group (T4) and the group fed only skim milk (T1). *In vitro* study indicated that both the strains were found to survive at pH 2.0 and 3.0 even up to 3 h and tolerate bile up to 2.0% concentration even after 12 h of exposure.

**Conclusions:**

This study revealed that oral administration of either *B. coagulans* B37 or *B. pumilus* B9 strains might be useful in reducing coliform counts accompanied by concurrent increase in lactobacilli counts in the intestinal flora in rats.

## Introduction

Probiotics are defined by the World Health Organization as “live microorganisms which, when administered in adequate amounts, confer a health benefit on the host” [1]. The global trend in the food sector is to incorporate probiotic microorganisms into foods for imparting its functional attributes that can provide some health-promoting component(s) beyond its traditional nutrients [2]. Worldwide, there are numerous strains of probiotics used in dietary supplements and foods, but most are unstable at room temperature and need to be freeze dried or encapsulated via special processes to remain viable during manufacturing, storage, and exposure to stomach acid and bile [3]. Lactic acid bacteria (LAB) have been used extensively as starter cultures in food fermentation and found to have many health benefits [4]. In recent years, research, development, and use of spore-forming bacteria as probiotic have brought a breakthrough in probiotic world.

The use of bacterial spore formers, *Bacillus* spp. in particular, as probiotics could provide practical advantages for both humans and animals [5]. The ability to form spores endows probiotics with higher resistance to technological stresses during production and storage processes and also higher resistance to gastric (pH, digestive enzymes) and intestinal environmental conditions [6]. Indeed, a number of products containing spores of *Bacillus* spp. are currently commercialized as probiotics for human [7] as well as for farm animals, particularly in cattle [8], swine [9], and poultry [10]. Besides many benefits, such as reduction of irritable bowel syndrome in human patients [11,12] and *Bacillus subtilis* is a use for vaccine production host and delivery vector as it is safe for human consumption and the production of spores exerting adjuvant effects [13].

There is limited information on the antimicrobial activity of *Bacillus probiotics* [14] *in vivo* animal model. Hence, we have conducted a study with the objectives (i) to elucidate the antimicrobial effect of *Bacillus coagulans* B37 and *Bacillus pumilus* B9 strains on fecal coliforms, (ii) to investigate their effect on fecal *Lactobacillus* as well as *Bacillus* spp. in rat animal model, and (iii) to examine functional potential in regards to acid and bile tolerance of these *Bacillus* strains under *in vitro* conditions.

## Materials and Methods

### Ethical approval

An experiment was designed to investigate the effect of *Bacillus* strains on fecal coliforms, *Lactobacillus* and *Bacillus* count in rat animal model at the National Dairy Research Institute, Karnal, Haryana, India. The experimental animals were treated in accordance with the guidelines of the Ethical Committee for Animal Experiments of National Dairy Research Institute.

### Bacillus strains for the feeding trail

*B. coagulans* B37 and *B. pumilus* B9 strains used for oral administration in rats in the current study were isolated from buffalo milk and subsequently characterized morphologically, biochemically, and genetically based on single strand conformational polymorphism banding patterns and partial 16S rRNA gene sequencing and also found on hemolysis on blood agar plates after incubation at 37°C for 18 h as previously reported [15]. Both *Bacillus* strains were maintained on *B. coagulans* agar (BCA) slants [16] and subcultured after every 25-30 days period. The stocks of both *Bacillus* strains were preserved in 20% glycerol stock medium at −76°C. The cultures were activated before use by twice subculturing in BCA broth. The spore biomass of *B. coagulans* B37 and *B. pumilus* B9 was produced using a whey-based medium supplemented with suitable nutrients and minerals [17].

### Experimental animals

36 adult male albino Wister rats were divided equally into to four groups (9 rats in each group) on the basis of their body weight and age so that the mean body weight and mean age of four groups did not differ (p>0.10) at the beginning of the experiment. Each group having 9 animals were again divided into three subgroups (3 animals in each subgroup) as replicates. The animals were 75±5 days old with mean body weight of 210±10 g. The animals were housed in aluminum cages under controlled temperature (22°C±2°C) and humidity (56±5%) at Small Animal House of National Dairy Research Institute, Karnal, Haryana, India. Each group having 9 animals were again divided into three subgroups (3 animals in each subgroup) as replicates.

### Feeding schedule

The experimental rats were allowed a 7-day adjustment period to remove the effect of stress possibly experienced by the animals due to separation from the main stock and to become accustomed to the testing regimen. All groups were fed on standard basal diet (containing starch - 57.46%, casein - 20.0%, sucrose - 5.0%, vegetable oil - 10.0%, cellulose powder - 1.91%, mineral mixture - 4.62%, and vitamin mixture - 1.01%) 7 days (adaptation period) before and 14 days after the treatment period. During the experimental period, the rats were fed twice, at morning (08:00 h) and evening (20:00 h) at 12 h interval. After the adaptation period, one group (T1) was fed on sterile skim milk along with basal diet for the next 28 days. The two experimental groups (T2 and T3) received spore biomass of *B. coagulans* B37 and *B. pumilus* B9, respectively, suspended in sterilized skim milk at 8-9 log colony-forming unit (cfu)/ml for 28 days as the previous report indicated that oral administration of a proprietary preparation of *B. coagulans -* GanedenBC(30)™ - a novel probiotic up to 9.38×10 (10) cfu/day for 1-year period caused no signs of toxicity in the parental generation (male or female) nor the F1 offspring of Wister rats [18]. No water was supplied to these three groups. After the feeding of *Bacillus* organisms for 28 days, all the animals were fed on the basal diet along with water for further 14 days. The last 14 days was considered as post-treatment period. The fourth group (T4) was supplied with clean water along with basal diet during the whole 49-day experimental period. This group served as control group.

### Fecal sample collection

The fecal samples were collected from all experimental rats to evaluate the concentration of bacterial spores. The fecal samples were collected at every 7-day interval starting from 0 to 49 days. A total of 288 fecal samples (8 fecal collections per rat) were collected in sterile container squeezing of the rectal area of the rat. Just after collection, the samples were weighed and immediately diluted in phosphate buffered saline (PBS) and then transported immediately in the laboratory and kept at 4°C in a refrigerator until analysis, performed on the same day. The fecal samples were pooled from 3 animals of each subgroup to obtain a total of 12 subgroups (3 from each group of T1, T2, T3, and T4). Each subgroup’s fecal sample was homogenized in PBS, and then, serial dilutions were made in the sterile physiological saline solution, and thereafter, subjected to enumeration of bacterial counts.

### Bacterial counts

All bacterial enumerations were done within 2 h of sampling. The homogenized samples in PBS were used for enumerating coliforms and lactobacilli counts using Eosin Methylene Blue agar and MRS agar (named by its inventors: DeMan, Rogosa, and Sharpe), respectively. For enumeration of *Bacillus* spore count in fecal samples, the homogenized samples in PBS were put into a water bath at 80°C for 10 min, and then cooled immediately to favor spore germination. The samples were serially diluted in physiological saline after holding 2 h at 37°C, and plating was performed using BCA media and incubated aerobically at 37°C for 24-72 h after which the *Bacillus* spores were identified on the basis of their characteristic morphology [19] and then counted and recorded.

### In vitro acid and bile tolerance test

The acid and bile tolerance of *B. coagulans* B37 and *B. pumilus* B9 strains were assessed as per the methods described previously [20,21]. Different low pH solutions were prepared to stimulate gastric acidic conditions by adding reagent grade HCl (35.8%) in distilled water drop by drop in a beaker and solutions having pH of 1.0, 2.0, and 3.0 were obtained. Sterile distilled water (pH adjusted to 6.5) was served as control. High bile salt solutions (1.0% and 2.0%) were prepared by dissolving bile salts (Hi-media, India) in distilled water. 1 ml of fresh culture containing approximately 10^7^-10^8^ cfu/ml was added to the different pH solutions (1.0, 2.0, 3.0, and 6.5) as well as bile solutions (1.0% and 2.0%) and mixed thoroughly. 1 ml of each solution was taken from each tube immediately (0 h) and 10-fold serial dilutions were prepared in 0.1% peptone water. Pour plating was done on BC agar media. The inoculated pH solutions were incubated at 37°C for 3 h, and 1 ml of culture was taken hourly from each tube after an interval of 1, 2, and 3 h of incubation followed by plating. The bile solutions containing cultures were incubated at 37°C for 12 h, and 1 ml of bile solution containing culture was taken from each tube after 1, 3, and 12 h of incubation and plated on BCA. All plates were incubated at 37°Cfor 24-72 h and the cfu were counted.

### Statistical analysis

T-test was employed to test the significance of differences between various groups of animals (rats) on age and body weight at the beginning of the experiment using Microsoft^®^ Excel 2000 Software package, Microsoft Corporation, USA. General Linear Model (GLM) procedure with post-hoc test was also done on each set of data of fecal *Coliform*, *Lactobacillus*, and *Bacillus* count to determine whether significant differences existed among different groups from SYSTAT 6.0.1 Statistical Software Package, 1996, SPSS, Inc., USA. Fisher’s least significant difference test was applied to determine the statistical significance of the difference in effects of treatments among different groups in the animal study, from matrix of pairwise comparison probabilities of different means. A p<0.05 or <0.01 was considered to be statistically significant. The mean±standard error of mean different parameters studied was graphically presented using GraphPad Prism 3.02, 1999, GraphPad Software Inc., San Diego CA.

## Results and Discussion

The present investigation was undertaken to explore the antibacterial effect of *B. coagulans* B37 and *B. pumilus* B9 on fecal coliform, *Lactobacillus*, and *Bacillus* in rat animal model. Despite the information on the antimicrobial effect of probiotic *Lactobacillus* and *Bifidobacterium* [22-24], there is a dearth of knowledge on the antimicrobial effect of *Bacillus* spp. in animal models [25].

### Effects on fecal coliform count

**Figure-1:**
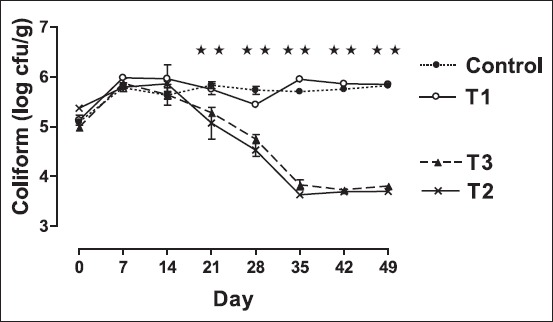
Trend in mean±standard error of mean fecal coliform count (colony-forming units/g fecal sample) in different groups of rat. Rats were fed with cholesterol-enriched diet from 0 up to 35^th^ day cholesterol-enriched diet. The diets were supplemented with control - water (•); T1 - Skim milk (○); T2 - non-fermented skim milk with B37 isolate (×); T3- non-fermented skim milk with B9 isolate from 15^th^ day up to 35^th^ day (▴). ⋆⋆indicates fecal coliform count among different groups differ significantly (p<0.01).

The effect of feeding of *B. coagulans* B37 and *B. pumilus* B9 on changes in the counts of fecal coliforms is presented in Figure-1. The number of fecal coliform counts decreased by more than 1 log cycle (p<0.01) for rats in both the groups received non-fermented skim milk supplemented with *Bacillus* organisms than the counts for control rats as well as the rats received only skim milk (T1) during the 49-day experimental period. However, the fecal coliform counts between the two groups fed *Bacillus* organisms did not differ (p>0.05). Fecal coliform counts for the control (T4) and the rats fed skim milk (T1) remained unchanged during the experimental period. In the present study, the feeding of *Bacillus* spores decreased the counts of fecal coliform in rats as reported previously in piglets [26]. Bacillus probiotic mixtures might be allowed for tailoring strategies to prevent infectious diseases [27,28]. Our earlier study showed the surface hydrophobicity of *B. coagulans* B37 and *B. pumilus* B9 were 42.88% and 37.43%, respectively, under *in vitro* condition [29]. In the present study, feeding of *Bacillus* organisms probably exerts an antagonistic effect on intestinal coliform leading to displacement of a considerable population of coliforms from the intestinal flora.

### Effect on fecal Lactobacillus count

**Figure-2:**
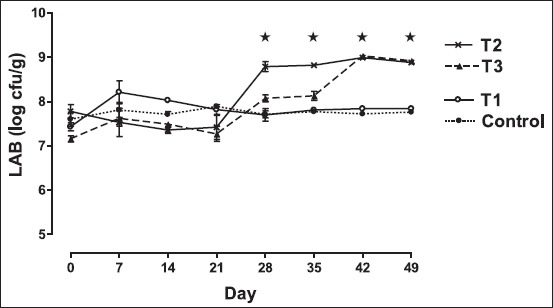
Trend in mean±standard error of mean fecal lactic acid bacteria (LAB) count (colony-forming units/g fecal sample) in different groups of rat. Rats were fed with cholesterol-enriched diet from 0 up to 35^th^ day cholesterol-enriched diet. The diets were supplemented with control - water (•); T1 - skim milk (○); T2 - non-fermented skim milk with B37 isolate (×); T3 - non-fermented skim milk with B9 isolate from 15^th^ up to 35^th^ day (▴). Fecal LAB count among different groups differ significantly (p<0.05). ⋆Indicates fecal lactobacilli count among different groups differ significantly (p<0.05).

Figure-2 shows the effect of dietary treatment of *Bacillus* organisms on changing pattern in the counts of fecal lactobacilli. The rats in the group fed non-fermented skim milk plus *B. coagulans* B37 exhibited significantly increase (p<0.05) in the numbers of lactobacilli (more than 1 log cycle) in their feces than that of the other groups during the 49-day experimental period. However, rats those received non-fermented milk along with *B. pumilus* B9 (T3) showed significantly higher (p<0.05) fecal lactobacilli counts than had the control group and the group fed only skim milk (T1) at day 42 and 49 also. The present result also depicted that there was no variation (p>0.05) in fecal lactobacilli counts between the two groups fed *Bacillus* probiotic at 42 and 49 days. The present finding of increase in the count of fecal lactobacilli agrees well with the earlier observations reflected large bowel colonization of LAB for biological effects [10,30,31]. Probably, the *Bacillus* organisms act synergistically with lactobacilli in the gut of rats, by producing acid and other metabolites essential for the growth of the lactobacilli.

### Effect on fecal Bacillus count

**Figure-3:**
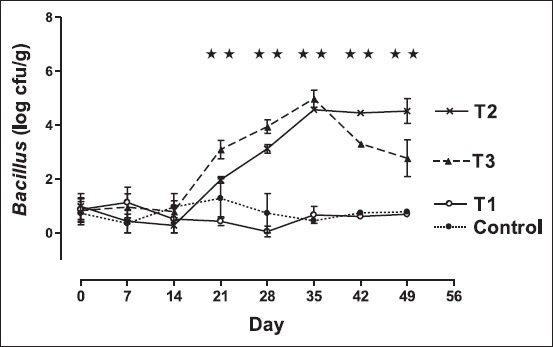
Trend in mean±standard error of mean fecal *Bacillus* count (colony-forming units/g fecal sample) in different groups of the rat. Rats were fed with cholesterol-enriched diet from 0 up to 35^th^ day cholesterol-enriched diet. The diets were supplemented with control - water (•); T1 - skim milk (○); T2 - non-fermented skim milk with B37 isolate (×); T3 - non-fermented skim milk with B9 isolate from 15^th^ day up to 35^th^ day (▴). ⋆⋆indicates fecal *Bacillus* count among different groups differ significantly (p<0.01).

The effect of feeding of *Bacillus organisms* on changing pattern in the counts of fecal *Bacillus* is presented in Figure-3. The fecal *Bacillus* counts in rats fed non-fermented skim milk plus *B. coagulans* B37 (T2) as well as rats fed non-fermented skim milk plus *B. pumilus* B9 (T3) increased immediately just after day 14 (days when the feeding of *Bacillus* organisms was started). The rate of increment was higher in group T3 than that of group T2. Average of 4 log cycle increment was evidenced on the 35^th^ day in both cases. As the probiotic feeding was stopped from 36^th^ day onward, the fecal spore count of group T2 decreased sharply within 7 days by more than 1 log cycle. However, the fecal *Bacillus* counts in groups T2 remained quite stable in post-feeding period. The *Bacillus* counts in feces of the control rats as well as the rats fed only skim milk (T1) were found to be very low or sometimes non-detectable. The significant increment of the *Bacillus* counts after feeding of *B. coagulans* B37 and *B. pumilus* B9 in rats strongly supports the previous reports on use of gut-colonizing *B. subtilis* spores as a new platform for the mucosal delivery of vaccine antigens [32] as well as survivability of *Bacillus clausii* spores in the human gastrointestinal (GI) tract, and thereafter germination, outgrowth, and multiplication as vegetative forms following oral administration as spore-based probiotic formulation [33]. In the present study, the feeding of *B. coagulans* B37 revealed that the fecal *Bacillus* count remained stable for 10 days after withdrawal of feeding of *B. coagulans* B37. This finding suggests that *B. coagulans* B37 probably adhere to the intestine of the rat and help to exert beneficial effects.

### In vitro acid and bile tolerance

Surviving passage through the GI tract is believed to be important for probiotics to function in the gut [34,35]. *In vitro* acid and bile tolerance abilities of the selected Bacillus isolates are presented in Tables-1 and 2. In the present study, both *B. coagulans* B37 and *B. pumilus* B9 strains were shown to survive at pH 2.0 and 3.0 even up to 3 h under *in vitro* experiment. *B. pumilus* B9 strain, specially, could survive at pH 1.0 up to 3 h, but *B. coagulans* B37 strain showed tolerance at pH 1.0 up to only 1 h. Resisting exposure to pH 3.0 for 1.5 to 2 h is considered one standard for low pH tolerance of probiotic bacteria [36]. Surviving bacteria from the stomach would then contact bile in the small intestine. Multiplication of *B. coagulans* in the small intestine should not be expected since the bacteria are strictly aerobic. Tolerance to bile is thus a criterion for *B. coagulans* to survive passage through the small intestine. Both *B. coagulans* B37 and *B. pumilus* B9 strains were found to possess a strong tolerance to bile up to 2.0% concentration even after 12 h of exposure. The significantly higher (p<0.01) counts of *Bacillus* in feces of rats fed with *Bacillus* spores than the counts in feces in rats that did not receive *Bacillus* spores, supports the previous finding of potential probiotic functions of *Bacillus* Species under simulated GI condition [37]. Hence, the present study suggests that consumption of non-fermented milk plus either *B. coagulans* B37 or *B. pumilus* B9 strains might be useful in reducing coliform counts accompanied by concurrent increase in lactobacilli counts in the intestinal flora in rats.

**Table-1:** *In vitro* acid tolerance of the selected *Bacillus* isolates.

Isolate	Bacterial count (cfu/ml)															

	At pH 6.4				At pH 1.0				At pH 2.0				At pH 3.0			
			
	0 h	1 h	2 h	3 h	0 h	1 h	2 h	3 h	0 h	1 h	2 h	3 h	0 h	1 h	2 h	3 h
*Bacillus coagulans* (B37)	8.64	8.11	8.34	8.49	3.69	2.23	-	-	6.18	5.85	5.28	5.36	6.81	6.73	6.53	6.11
*Bacillus pumilus* (B9)	8.86	8.79	8.18	7.96	6.32	5.67	3.51	2.11	7.86	6.36	6.23	5.85	7.96	7.59	7.18	6.87

cfu=Colony-forming units

**Table-2:** *In vitro* bile tolerance of the selected *Bacillus* isolates.

Isolate	Bacterial count (cfu/ml)											

	At 0.0% bile				At 1.0% bile				At 2.0% bile			
		
	0 h	1 h	3 h	12 h	0 h	1 h	3 h	12 h	0 h	1 h	3 h	12 h
*Bacillus coagulans* (B37)	8.15	7.60	8.15	7.95	7.46	6.11	6.86	6.81	6.28	5.62	5.48	5.53
*Bacillus pumilus* (B9)	8.75	8.15	7.99	7.85	6.43	5.95	5.64	6.28	5.70	5.36	4.59	4.87

cfu=Colony-forming units

## Conclusions

As *Bacillus* spp. is not considered resident members of the gut microflora, controversies are there whether it can survive the transit within GI tract and can be able to exert health protecting effects followed by germination of spores within the gut. *In vivo* as well *as* *in vitro* study clearly indicated that *B. coagulans* B37 and *B. pumilus* B9 spores could withstand the adverse environment of the GI. tract. The decreasing counts in fecal coliform with the increasing counts in both lactobacilli and *Bacillus* counts in the feces of the treated rats suggested that both *B. coagulans* B37 and *B. pumilus* B9 probably inhibited the proliferation of coliform in rats and exerted synergistic effects with lactobacilli. Thus, both *B. coagulans* B37 and *B. pumilus* B9 could be used as an adjunct probiotic in combination with established strains of probiotic lactobacilli to achieve combined and more pronounced health-promoting effects. Since Food and Drug Administration has some restrictions for granting GRAS (Generally Regarded as Safe) status for any *Bacillus* species, two indigenous strains, *B. coagulans* B37 and *B. pumilus* B9 could be considered for probable probiotics after necessary clinical studies and safety assessment in animals and human.

## Authors’ Contributions

Both the authors contributed to conception and design of the study. LH carried out the research work, collected data, analyzed data, interpreted the results, and drafted the article critically for important intellectual content. DNG monitored the whole research program. Both authors have read and approved the final manuscript.

## References

[1] FAO/WHO (2002). Guidelines for the Evaluation of Probiotics in Food. Report of a Joint FAO/WHO Working Group on Drafting Guidelines for Evaluation of Probiotics in Food. London, Ontario, Canada. April 30 and May 01, 2002.

[2] Butel M.J (2014). Probiotics, gut microbiota and health. Med. Mal. Infect.

[3] Martinez R.C, Bedani R, Saad S.M (2015). Scientific evidence for health effects attributed to the consumption of probiotics and prebiotics:An update for current perspectives and future challenges. Br. J. Nutr.

[4] Kumari A, Catanzaro R, Marotta F (2011). Clinical importance of lactic acid bacteria:A short review. Acta Biomed.

[5] Cutting S.M (2011). Bacillusprobiotics. Food Microbiol.

[6] Bader J, Albin A, Stahl U (2012). Spore-forming bacteria and their utilisation as probiotics. Benef. Microbes.

[7] Lefevre M, Racedo S.M, Ripert G, Housez B, Cazaubiel M, Maudet C, Jüsten P, Marteau P, Urdaci M.C (2015). Probiotic strain *Bacillus* subtilis CU1 stimulates immune system of elderly during common infectious disease period:A randomized, double-blind placebo-controlled study. Immun. Ageing.

[8] Peng H, Wang J.Q, Kang H.Y, Dong S.H, Sun P, Bu D.P, Zhou L.Y (2012). Effect of feeding *Bacillus subtilis* natto fermentation product on milk production and composition, blood metabolites and rumen fermentation in early lactation dairy cows. J. Anim. Physiol. Anim. Nutr. (Berl).

[9] Larsen N, Thorsen L, Kpikpi E.N, Stuer-Lauridsen B, Cantor M.D, Nielsen B, Brockmann E, Derkx P.M, Jespersen L (2014). Characterization of *Bacillus* spp. Strains for use as probiotic additives in pig feed. Appl. Microbiol. Biotechnol.

[10] Jeong J.S, Kim I.H (2014). Effect of *Bacillus subtilis* C-3102 spores as a probiotic feed supplement on growth performance, noxious gas emission, and intestinal microflora in broilers. Poult. Sci.

[11] Urgesi R, Casale C, Pistelli R, Rapaccini G.L, de Vitis I (2014). A randomized double-blind placebo-controlled clinical trial on efficacy and safety of association of simethicone and *Bacillus coagulans* (Colinox®) in patients with irritable bowel syndrome. Eur. Rev. Med. Pharmacol. Sci.

[12] Choi C.H, Kwon J.G, Kim S.K, Myung S.J, Park K.S, Sohn C.I, Rhee P.L, Lee K.J, Lee O.Y, Jung H.K, Jee S.R, Jeen Y.T, Choi M.G, Choi S.C, Huh K.C, Park H (2015). Efficacy of combination therapy with probiotics and mosapride in patients with IBS without diarrhea:A randomized, double-blind, placebo-controlled, multicenter, Phase II trial. Neurogastroenterol Motil.

[13] Rosales-Mendoza S, Angulo C (2015). *Bacillus subtilis* comes of age as a vaccine production host and delivery vehicle. Exp. Rev. Vaccines.

[14] Raut S.V, Pingle Y.A (2010). Screening and characterization of antimicrobial substances produced by *Bacillus* species. J. Pure Appl. Microbiol.

[15] Haldar L, Gandhi D.N, Majumdar D, De S (2015). Characterization of indigenous *Bacillus coagulans* isolated from cattle and buffalo milk. Int. J. Microbiol. Res.

[16] Atlas R.M (2004). Handbook of Microbiological Media.

[17] Rana R, Gandhi D.N (2000). Effect of basal medium and pH on the growth of *Lactobacillus acidophilus*. Indian J. Dairy Sci.

[18] Endres J.R, Qureshi I, Farber T, Hauswirth J, Hirka G, Pasics I, Schauss A.G (2011). One-year chronic oral toxicity with combined reproduction toxicity study of a novel probiotic *Bacillus coagulansas* a food ingredient. Food Chem. Toxicol.

[19] De Clerck E, Rodriguez-Diaz M, Forsyth G, Lebbe L, Logan N.A, De Vos P (2004). Polyphasic characterization of *Bacillus coagulans* strains, illustrating heterogeneity within this species, and emended description of the species. Syst. Appl. Microbiol.

[20] Clark P.A, Cotton L.N, Martin J.H (1993). Selection of *Bifidobacteria* for use as dietary adjuncts in cultured dairy foods:II. Tolerance to simulated pH of human stomachs. Cult. Dairy Prod. J.

[21] Clark P.A, Martin J.H (1994). Selection of *Bifidobacteria* for use as dietary adjuncts in cultured dairy foods:III. Tolerance to simulated bile concentrations of human small intestines. Cult. Dairy Prod. J.

[22] de Oliveira C.P, da Silva J.A, de Siqueira-Júnior J.P (2015). Nature of the antimicrobial activity of *Lactobacillus casei, Bifidobacterium bifidum* and *Bifidobacterium animalis* against foodborne pathogenic and spoilage microorganisms. Nat. Prod. Res.

[23] Georgieva R, Yocheva L, Tserovska L, Zhelezova G, Stefanova N, Atanasova A, Danguleva A, Ivanova G, Karapetkov N, Rumyan N, Karaivanova E (2015). Antimicrobial activity and antibiotic susceptibility of *Lactobacillus* and *Bifidobacterium* spp. Intended for use as starter and probiotic cultures. Biotechnol. Biotechnol. Equip.

[24] Mazaya B, Hamzawy M.A, Khalil M.A, Tawkol W.M, Sabit H (2015). Immunomodulatory and antimicrobial efficacy of *Lactobacilli* against enteropathogenic infection of *Salmonella typhi*: *In-vitro* and *in-vivo* study. Int. J. Immunopathol. Pharmacol.

[25] Vidya Laxme B, Rovetto A, Grau R, Agrawal R (2014). Synergistic effects of probiotic *Leuconostoc mesenteroides* and *Bacillus subtilis* in malted ragi (*Eleucine corocana*) food for antagonistic activity against V. Cholerae and other beneficial properties. J. Food Sci. Technol.

[26] Tsukahara T, Tsuruta T, Nakanishi N, Hikita C, Mochizuki M, Nakayama K (2013). The preventive effect of *Bacillus subtilus* strain DB9011 against experimental infection with enterotoxcemic *Escherichia coli* in weaning piglets. Anim. Sci. J.

[27] Lin Z, Shi Y, Deng B, Mao X, Yu D, Li W (2015). Protective immunity against *Eimeria tenella* infection in chickens following oral immunization with *Bacillus subtilis* expressing *Eimeria tenella* 3-1E protein. Parasitol. Res.

[28] Zhou D, Zhu Y.H, Zhang W, Wang M.L, Fan W.Y, Song D, Yang G.Y, Jensen B.B, Wang J.F (2015). Oral administration of a select mixture of *Bacillus* probiotics generates Tr1 cells in weaned F4ab/acR - Pigs challenged with an F4+ETEC/VTEC/EPEC strain. Vet. Res.

[29] Haldar L, Gandhi D.N, Mazumdar D (2016). Functional and probiotic potential of indigenous *Bacillus coagulans* and *Bacillus pumilus* strains isolated from buffalo milk. Int. J. Microbiol. Res.

[30] Donnet-Hughes A, Rochat F, Serrant P, Aeschlimann J.M, Schiffrin E.J (1999). Modulation of nonspecific mechanisms of defense by lactic acid bacteria:Effective dose. J. Dairy Sci.

[31] Hosoi T, Ametani A, Kiuchi K, Kaminogawa S (2000). Improved growth and viability of lactobacilli in the presence of *Bacillus subtilis*(natto), catalase, or subtilisin. Can. J. Microbiol.

[32] Tavares Batista M, Souza R.D, Paccez J.D, Luiz W.B, Ferreira E.L, Cavalcante R.C, Ferreira R.C, Ferreira L.C (2014). Gut adhesive *Bacillus subtilis* spores as a platform for mucosal delivery of antigens. Infect. Immun.

[33] Ghelardi E, Celandroni F, Salvetti S, Gueye S.A, Lupetti A, Senesi S (2015). Survival and persistence of *Bacillus clausii* in the human gastrointestinal tract following oral administration as spore-based probiotic formulation. J. Appl. Microbiol.

[34] Baick S.C, Kim C.H (2015). Assessment of characteristics and functional properties of *Lactobacillus* species isolated from kimchi for dairy use. Korean J. Food Sci. Anim. Resour.

[35] Hanifi A, Culpepper T, Mai V, Anand A, Ford A.L, Ukhanova M, Christman M, Tompkins T.A, Dahl W.J (2015). Evaluation of *Bacillus subtilis* R0179 on gastrointestinal viability and general wellness:A randomised, double-blind, placebo-controlled trial in healthy adults. Benef. Microbes.

[36] Fontana L, Bermudez-Brito M, Plaza-Diaz J, Muñoz-Quezada S, Gil A (2013). Sources, isolation, characterisation and evaluation of probiotics. Br. J. Nutr.

[37] Shobharani P, Halami P.M (2014). Cellular fatty acid profile and H(+)-ATPase activity to assess acid tolerance of *Bacillus* sp. For potential probiotic functional attributes. Appl. Microbiol. Biotechnol.

